# Determinants of protein evolutionary rates in light of ENCODE functional genomics

**DOI:** 10.1186/1471-2105-15-S3-A9

**Published:** 2014-02-11

**Authors:** Nadezda Kryuchkova, Marc Robinson-Rechavi

**Affiliations:** 1 Department of Ecology and Evolution, University of Lausanne, 1015 Lausanne, Switzerland; 2 Swiss Institute of Bioinformatics (SIB), 1015 Lausanne, Switzerland

## Background

The influence of different parameters, from gene size to expression levels, on the evolution of proteins has been previously studied mostly in yeast [1] and *Drosophila *[2]. The main feature which has been found to explain protein evolutionary rate was the level of gene expression, especially in yeast.

## Results

**Figure 1:**
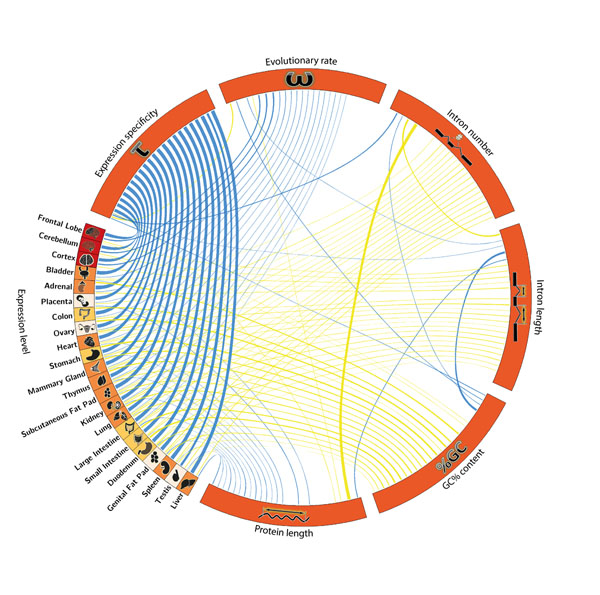
Partial correlations of gene parameters (mouse data) with Pearson correlation coefficient. The width of connections shows the strength of the correlations. Only correlations with p-value > 0.001 are shown. Positive correlations are represented in yellow and negative in blue. Tissues were ordered according to correlation with evolutionary rate.

Here we investigate these relations further, and extend them to mammals, especially taking in account gene expression in different organs. For expression we used the RNA-seq data from ENCODE [3] for 22 different tissues of mouse. We used ENCODE data to define which transcript is used as reference to compute features such as gene length or intron number. The relation between evolutionary rate and six gene features: gene expression, gene expression specificity, intron number, intron length, protein length and GC% content were analyzed. We use partial correlation to take into account dependencies between them. We find strong differences between tissues in the impact of expression on evolutionary rate (Figure 1 and http://f1000.com/posters/browse/summary/1094165). Over all tissues, an interesting result is that evolutionary rate shows no strong correlation with expression level in mouse if corrected for other parameters.

## Conclusions

Dependencies between gene features need to be taken into account for an unbiased view of gene evolution. Overall results are consistent with those in *Drosophila *[2]. We find important differences between tissues in the relation between expression and evolutionary rate, especially for the central nervous system and testis.
